# Owner-reported prevalence and severity of fear and anxiety in dogs

**DOI:** 10.1007/s11259-026-11227-y

**Published:** 2026-04-25

**Authors:** Bonnie V. Beaver

**Affiliations:** Department of Small Animal Clinical Sciences College of Veterinary Medicine & Biomedical Sciences, Texas A&M University, College Station, 77843 TX USA

**Keywords:** Dog, Anxiety, Fear, Aggression

## Abstract

Anxiety and fear in animals affect their current, and potential long-term, welfare. The prevalence of this problem has been studied by various authors, but considerable variety exists in reported values, methods used to gather data, and location of the dogs. Because preferred breeds and the way dogs are raised and kept vary considerably by country, the location of the animals studied can affect reported data. In order to obtain robust estimates in this regard for the United States, data from 43,517 dogs enrolled by their owners in the Dog Aging Project (DAP) were used to determine the prevalence and severity of anxiety/fear shown by these dogs. Behaviors of anxiety and fearfulness were assessed based on owners’ responses to nine questions. These questions were divided into four multi-question categories (anxiety/fearfulness in response to unfamiliar people, unfamiliar dogs, unfamiliar occurrences in the environment, and grooming). Ratings used by owners were based on a 5-point scale between 0 (no problem) and 4 (extreme fear). Data were analyzed to determine the prevalence of this problem at each rating level. Overall, 91.0% of dogs in the study received at least one rating of level 2 (mild/moderate fear/anxiety) or higher in severity across all nine questions. Specifically, the studied dogs were reported to show fear/anxiety toward unfamiliar people (22.3% of dogs); unfamiliar dogs (47.4%); unfamiliar situations, objects, or noise (25.5%); and grooming (33.0%).

## Introduction

Fear and anxiety are related emotions that can be associated with a variety of situations considered threatening by a dog. Physiologically, most anxiety is short-term, lasting minutes or hours, but an animal’s health can be negatively affected when anxiety becomes stressful and is experienced over long periods of time.

Several canine behavior studies included anxiety or fear in the information (Martínez et al. 2011; Col et al. 2016; Yamada et al. 2019; Dinwoodie et al. 2019; Salonen et al. 2020; Anderson et al. 2022). Unfortunately, data between studies is quite variable, possibly because the studies were based in different countries, data was sourced from behavior practice patients or public surveys, data gathering techniques and questions differed, and results were presented in different ways. For example, general fearfulness was diagnosed in dog behavior cases seen in behavior specialty practices ranged from 3.8% to 30.2% (Khoshnegah et al. 2011; Martínez et al. 2011; Col et al. 2016; Anderson et al. 2022; Meyer et al. 2023). These practices were located in Australia, Spain, Denmark, United States, and Iran. Results from survey questionnaires available to the dog-owning public suggest fearful behaviors are even more variable. Publications based on surveys in Finland and Japan report the prevalence of general fearfulness as between 20% and 26% (Tiira et al. 2016; Salonen et al. 2020); but other reports suggest the prevalence of fear is considerably higher, from 44% to near 90% (Dinwoodie et al. 2019; Powell et al. 2021).

The Dog Aging Project (DAP), a community science study, was designed to collect extensive data about dogs in the United States over several years (Creevy et al. 2022). The value of using DAP data lies in the immensity of the data pool—43,517 dogs at the time of this study. The topics of information gathered from dog owners include dog health, diet, activity, source, environments, and behavior. Behavioral data collected in the DAP is based on responses to the previously validated Canine Behavioral Assessment and Research Questionnaire (C-BARQ) (Hsu and Serpell 2003). Dr. James Serpell granted permission to use and facilitated validation of the nine-question shortened version for use in the DAP (Canine Behavioral Assessment & Research Questionnaire (short version) 2019; The C-BARQ Questionnaire 2021; Wilkins et al. 2024). Owners were given the following instructions for this section: “Dogs often show signs of anxiety or fear when exposed to particular sounds, objects, persons or situations (e.g., crouching or cringing with tail tucked between the legs; whimpering or whining, freezing; trembling; or attempting to escape or hide). Using the following 5-point scale (0 = No fear, 4 = Extreme fear), please indicate your own dog’s recent tendency to display fearful behavior in the following circumstances.” For each question, owners assessed their dog’s behavior level between 0 and 4. The rating of 0 represented no visible signs of anxiety or fearfulness. The rating of 2 represented mild/moderate signs, such as avoiding eye contact, avoiding the feared object, crouching or cringing with tail lowered or tucked between the legs, whimpering and whining, freezing, and shaking and trembling. A 4 rating represented extreme fear as shown by exaggerated cowering and/or vigorous attempts to escape, retreat, or hide from the object, person, or situation (Canine Behavioral Assessment & Research Questionnaire (short version) 2019; The C-BARQ Questionnaire^2021^). No specific descriptors were given for ratings of 1 or 3. Responses are intended to indicate the dog’s recent tendencies toward anxiety and fear in specific contexts and are not intended to be diagnostic.

Given the reported prevalence from published studies, anxiety in a large population of dogs deserves study, as the findings can aid in understanding its causes, determining when treatment is needed, and establishing management plans.

## Materials and methods

Permission was obtained to access the Dog Aging Project data, and the 2022 data were downloaded in an Excel spreadsheet. This contained extensive information about 43,517 dogs of various breeds, ages, sexes, sizes, and locations within the United States (Dog Aging Project, 2024).

Data from each of the 43,517 dogs was reviewed for each of the nine questions in the anxiety/fear category and then manually totaled by level for each question. Additionally, it was manually determined how many dogs were reported to have multiple responses at levels 2, 3 or 4 in total and in each of four groupings based on similarities of questions: fearful of strangers; strange dogs; unfamiliar noises, objects, or situations; and nail trims or baths. All dogs for which there was a rating were included. When more than one question was considered, only dogs with responses for all questions were included. In this study, dogs with ratings of 2 or greater were considered to have gradually increasing levels of anxiety or fear.

## Results

The questionnaire’s nine questions about anxiety/fear were grouped into four categories to permit comparisons between similar scenarios (Table 1). Of all dogs in the study, 39,574 (91.0%) had at least one rating of 2 (mild/moderate), 3, or 4 (extreme) across the nine questions, but only three dogs were rated as extreme for all nine questions.

**Table 1:** The number of dogs (and percentage of dogs responding) rated as by their owners relative to fear in response to questions about their recent behavior

Question	Number of responses	Levels 0 & 1	Level 2 mild/moderate	Level 3	Level 4 extreme
Fear responses toward unfamiliar people					
When approached directly by an unfamiliar person while away from your home	43,153	35,027 (81.2%)	5,900 (13.7%)	1,537 (3.6%)	690 (1.6%)
When an unfamiliar person tries to touch or pet the dog	43,265	35,711 (82.5%)	4,861 (11.2%)	1,758 (4.1%)	932 (2.2%)
Fear responses toward unfamiliar dogs					
When approached directly by an unfamiliar dog	43,075	31,425 (73.0%)	7.967 (18.5%)	2,595 (6.0%)	1,088 (2.5%)
When barked, growled, or lunged at by an unfamiliar dog	42,592	23,109 (54.3%)	11,969 (28.1%)	4,883 (11.5%)	2,631 (6.2%)
Fear responses toward unfamiliar noise, objects, or situations					
In response to sudden or loud noises	43,325	24,637 (56.9%)	9,788 (22.6%)	4,718 (10.9%)	4,182 (9.6%)
In response to strange or unfamiliar objects on or near the sidewalk	43,224	37,190 (86.0%)	4,675 (10.8%)	1,064 (2.5%)	395 (0.9%)
When first exposed to unfamiliar situations	43,200	30,394 (70.4%)	8,897 (20.6%)	2,738 (6.3%)	1,171 (2.7%)
Fear responses toward nail trims and baths					
When having nails clipped by a household member	41,167	25,840 (62.8%)	8,058 (19.6%)	3,796 (9.2%)	3,473 (8.4%)
When groomed or bathed by a household member	42,803	33,095 (77.3%)	6,519 (15.2%)	2,076 (4.9%)	1,113 (2.6%)

Because fear of nail trims and bathing are learned behaviors instead of instinctive ones, data was also evaluated without including results from those two questions. When responses to the two questions about fears of nail trims and grooming were omitted, 36,562 (84.0%) dogs had at least one rating of 2, 3, or 4 across the remaining seven questions. Of these dogs, 21,845 (59.7%) were reported as fearful on multiple questions and 977 were reported as fearful on all seven, 19 of which were extremely fearful (ratings of 4).

Across the two questions about fear of strangers, 33,571 (77.1%) of the 43,517 dogs did not show fear or anxiety (rating of 0 or 1). Another 3,936 dogs were rated with mild/moderate to extreme fear (ratings of 2, 3, or 4) for one question; 5,759 were rated 2, 3, or 4 for both questions, including 426 with extreme fear in both questions.

Of all dogs in the DAP study, 22,684 (52.1%) were rated as not anxious or fearful of unfamiliar dogs (ratings of 0 or 1). There were 10,129 dogs (23.3%) rated mildly/moderately fearful or anxious (ratings of 2–4) on one of the two questions. Another 10,502 dogs (24.1%) had ratings of 2, 3, or 4 for both questions, with 974 of them rated as extremely fearful.

According to the DAP survey, 19,192 (44.1%) dogs were rated by owners as not fearful (ratings of 0 or 1) to noise, strange objects, or new situations in separate questions. Another 14,611 dogs (33.6%) were rated as mild/moderate to extremely fearful (ratings of 2–4) for one of the three questions; 9,692 dogs (22.3%) described as having anxiety/fear at levels 2, 3, or 4 in at least two questions, with 82 of them rated as extremely fearful (rating of 4) in all three situations.

The last two questions had owners rate the dog’s tendency to show anxiety or fear when having their nails clipped or when bathed by members of the household. Across both questions, 15,432 dogs (35.5% of all dogs) were rated by owners as not being fearful of grooming (ratings of 0 or 1), and 6,840 dogs (15.7%) rated as mild/moderate to extremely fearful (ratings of 2–4) for only one of the questions. Another 7,507 dogs were rated as fearful at levels 2–4 to both questions, with 683 of them rated as extremely fearful for both types of grooming.

## Discussion

While most behaviorists and some owners consider responses rated as 2 to 4 in any of the categories studied to be problematic, it is important to recognize that not all owners consider a behavior to be problematic even at an extreme or severe level and that responses are likely appropriate for the situation. Reported fears of unfamiliar people and dogs probably represent mixed behavioral motivations with differing underlying causes. A previous study of 427 dogs, which used the same shortened version of the C-BARQ questionnaire, addressed how perceptions vary (Powell et al. 2021). This study compared responses from current pet owners to those from owners who had relinquished their pets. The former were more likely than the latter to indicate fearful behavior in their dogs (approximately 90% vs. approximately 78% respectively). These findings suggest that those who relinquish their dogs might be less tolerant of certain behaviors (Powell et al. 2021) and that current pet owners, being more tolerant, might not consider behavior rated as moderate to extreme to be a problem worthy of professional help.

In responses to each question, it was shown that most dogs are not or only mildly anxious, but they may have a higher level of fear in a few cases. This is reflected when considering the results across all nine DAP questions about anxiety and fear, which showed that 91.0% of dogs were rated as having mild/moderate to extreme levels of fear on at least one question. When questions addressing nail trims and baths were excluded, the percentage of affected dogs dropped to 84.0% anxious or fearful on at least one question.

According to the DAP data, 22.3% of dogs show at least mild/moderate anxiety toward unfamiliar people. This finding is consistent with the 15% to 21.4% results of five other surveys (Martínez et al. 2011; Tiira et al. 2016; Yamada et al. 2019; Dinwoodie et al. 2019; Salonen et al. 2020), but they are considerably lower than previously reported in another study that used the same short version of the C-BARQ used here (Powell et al. 2021).

A fearful response to unfamiliar dogs was more inconsistent than that toward unfamiliar people. Previous study results varied considerably from this study. The lowest prevalence of fearfulness toward unfamiliar dogs reported was 10% (Dinwoodie et al. 2019). Other studies ranged from 17% to 80.0%, even when they used the same questionnaire as did this study (Yamada et al. 2019; Solonen et al. 2020; Powell et al. 2021). The current study found that 47.4% of dogs showed some level of anxiety or fearfulness toward unfamiliar dogs.

Fear of noise arising from sharp, unpredictable sounds like thunder or firecrackers is a common problem (Martínez et al. 2011; Tiira et al. 2016; Salonen et al. 2020; Duxbury et al. 2023). Prevalence reports from behavioral specialty practices ranged up about 10% (Bamberger and Houpt 2006; Anderson et al. 2022). In contrast, prevalences found in surveys ranged from 26% to 51.7% (Segurson et al. 2005; Martínez et al. 2011; Tiira et al. 2016; Yamada et al. 2019; Salonen et al. 2020), which is more consistent with the prevalence of 42.9% found in this study. Of concern is that almost 10% of dogs in this study were rated with extreme noise fear, a level at which the problem becomes difficult to treat.

This study also found that 13.9% of owners rated their dog showing fear of new objects and 29.4% showing situational anxiety. Although the available literature contains no statistics for comparison regarding fear of objects, the prevalence found of situational fears found here is somewhat higher than the 17.9% previously reported (Tiira et al. 2016).

Fearful responses to grooming are thought to be common, and in this study, 35.2% of dogs were rated with at least a mild/moderate fear of nail trims and 22.3% showed at least a mild/moderate anxiety/fear of baths. Across both questions, 7,507 dogs (17.3% of all dogs) were rated at levels 2, 3, or 4, with 683 of them rated as extremely fearful on both questions. The only other report found of prevalence of problems with grooming (4%) is in a Danish survey (Meyer et al. 2023).

Some features of the C-BARQ versions complicate the interpretation of anxiety because questions that include signs suggestive of anxieties relating to separation from the owner are listed in different categories in the questionnaire—separation-related behavior and attachment/attention-seeking categories. Additionally, assessment was difficult because of the possible co-existence of fear and aggression, with aggression being in a separate category as well. Dogs can show several signs of fear, including aggression, but the DAP survey did not differentiate dogs that bite out of fear from those that were just fearful or just aggressive toward unfamiliar people or dogs. Behaviors that reflect anxiety/fear might have been put into the separation-related or aggression categories or in both categories. Without a physical description of the stimuli of the behavior, data can only be generalized by category.

## Conclusion

Of all dogs in the Dog Aging Project, 91% were rated by owners as at least moderately fearful in one or more of nine situations. When the two questions about grooming were omitted from analysis, the prevalence of anxiety/fear dropped to 84% of dogs. Less than 3% of all dogs were rated as extremely fearful across all questions.

## Data Availability

This research is based on publicly available data collected by the Dog Aging Project, which is supported by U19 grant AG057377 (PI: Daniel Promislow) from the National Institute on Aging, a part of the National Institutes of Health, and by additional grants and private donations. These data are housed on the Terra platform at the Broad Institute of MIT and Harvard and permission was obtained to assess the data.
